# Evaluation of Meropenem Pharmacokinetics in an Experimental Acute Respiratory Distress Syndrome (ARDS) Model during Extracorporeal Membrane Oxygenation (ECMO) by Using a PenP *β*-Lactamase Biosensor

**DOI:** 10.3390/s18051424

**Published:** 2018-05-04

**Authors:** Max Andresen, Joaquin Araos, Kwok-Yin Wong, Yun-Chung Leung, Lok-Yan So, Wai-Ting Wong, Salvador Cabrera, Camila Silva, Leyla Alegria, Alejandro Bruhn, Dagoberto Soto

**Affiliations:** 1 Laboratory 103B, Centro de Investigaciones Médicas, Departamento de Medicina Intensiva, Facultad de Medicina y Hospital Clínico, Pontificia Universidad Católica de Chile, Marcoleta 391, Santiago 8330024, Chile; juaccco@gmail.com (J.A.); cfsilvag2@gmail.com (C.S.); lalegria@med.puc.cl (L.A.); alejandrobruhn@gmail.com (A.B.); dasotom@uc.cl (D.S.); 2 State Key Laboratory of Chirosciences, Department of Applied Biology and Chemical Technology, The Hong Kong Polytechnic University, Hung Hom, Kowloon, Hong Kong, China; kwok-yin.wong@polyu.edu.hk (K.-Y.W.); thomas.yun-chung.leung@polyu.edu.hk (Y.-C.L.); lok-yan.so@polyu.edu.hk (L.-Y.S.); wai-ting.wong@polyu.edu.hk (W.-T.W.); 3 Departamento de Farmacia, Facultad de Farmacia, Universidad de Concepción, Víctor Lamas 1290, Concepción 4070386, Chile; scabrera@ssconcepcion.cl

**Keywords:** biosensor, PenP, pharmacokinetic, meropenem, ECMO

## Abstract

Introduction: The use of antibiotics is mandatory in patients during extracorporeal membrane oxygenation (ECMO) support. Clinical studies have shown high variability in the antibiotic concentrations, as well as sequestration of them by the ECMO circuit, suggesting that the doses and/or interval administration used during ECMO may not be adequate. Thus, a fast response sensor to estimate antibiotic concentrations in this setting would contribute to improve dose adjustments. The biosensor PenP has been shown to have a dynamic range, sensitivity and specificity useful for pharmacokinetic (PK) tests in healthy subjects. However, the use of this biosensor in the context of a complex critical condition, such as ECMO during acute respiratory distress syndrome (ARDS), has not been tested. Objectives: To describe, by using PenP Biosensor, the pharmacokinetic of meropenem in a 24-h animal ARDS/ECMO model. Methods: The PK of meropenem was evaluated in a swine model before and during ECMO. Results: The PK parameters such as maximum concentration (Cmax), elimination rate constant (Ke), and cleareance (Cl), were not significantly altered during ECMO support. Conclusions: (a) ECMO does not affect the PK of meropenem, at least during the first 24 h; and (b) PenP has the potential to become an effective tool for making medical decisions associated with the dose model of antibiotics in a critical patient context.

## 1. Introduction

Meropenem is a useful and frequent antibiotic choice for treating severe infections in critically ill patients in the intensive care unit (ICU) [1,2]. Since meropenem action is time-dependent, a careful administration and dosing plan should be made in order to maintain concentrations above the minimum inhibitory concentration (MIC) [3,4,5].

To add more to the complexity of meropenem administration, extracorporeal devices frequently used in critically ill patients can dramatically alter antimicrobial concentrations. Extracorporeal membrane oxygenation (ECMO) has rapidly expanded as a salvage strategy in ICU patients when respiratory support is required in cases of severe acute respiratory distress syndrome (ARDS) [6,7,8,9,10]. The use of antibiotics is mandatory in patients during ECMO support, thus it is essential to understand any potential changes in their pharmacokinetics (PK) to adjust dosage and to improve the clinical outcomes [11,12]. There are limited data to guide antibiotic therapy in adults receiving ECMO support, especially regarding carbapenems [13,14,15,16,17,18,19,20,21]. Meropenem plasmatic concentrations show high variability during ECMO and high sequestration rates of the antibiotic, with only 20% of the initial dose being detected in an isolated circuit [22]. Currently, the evaluation of the effectiveness of meropenem treatment during ARDS/ECMO is challenging, since there are no available methods for quickly assessing the success or failure of the dosages used. Thus, it is crucial to develop a technique that allows the clinician to approximate to the actual pharmacokinetics of this drug during ECMO.

Our group developed a method to quantify *β*-lactam antibiotics by using a biosensor based on a mutant of *β*-lactamase TEM-1, PenP. This novel method is easy to implement, fast, precise, sensitive and selective. Furthermore, the dynamic range of this biosensor has an amplitude of 2 log around the inflection point centered at log −6.2 ± 0.03. This dynamic range is at or below the MIC of the pathogens targeted by meropenem, making the biosensor a useful tool to determine clinically relevant information such as pharmacokinetics (PK) of the drug at the bedside. Indeed PenP has been shown to be useful for PK testing in animals and healthy humans [23,24]. Despite these promising results, the usefulness of this biosensor in situations of high complexity such as those observed in critically ill patients, has not yet been proven. The aim of our study was to describe the PK of meropenem in a complex animal (*Sus Scrofa*) model of ARDS/ECMO by using the fluorescent biosensor PenP.

## 2. Material and Methods

### 2.1. Chemicals and Reagents

Meropenem was obtained from a local supplier (Pharma Investi, Santiago, Chile). All other chemicals were purchased from Sigma-Aldrich (St Louis, MO, USA). Phosphate saline buffer (PBS): 8 g/L NaCl, 0.2 g/L KCl, 2.72 g/L Na_2_HPO_4_, and 0.24 g/L NaH_2_PO_4_, pH 7.0. Dilution buffer: 1% (*w*/*v*) BSA in PBS buffer (pH 7.0).

### 2.2. Animal Model

#### 2.2.1. Ethics

This study was conducted with the approval of the Pontificia Universidad Católica de Chile Animal Ethics Committee, approval number 12-029. Experiments were performed in accordance to the Guide for the Care and Use of Laboratory Animals, 8th Edition, from the National Academy of Sciences of the United States of America.

#### 2.2.2. Meropenem Infusion and Blood Sampling

Five adult pigs (*Sus Scrofa*, 29.3 ± 0.95 Kg) were infused with intravenous meropenem and then blood samples were drawn in order to construct PK curves. Meropenem were reconstituted in saline solution (500 mg/100 mL) and subsequently infused over 30 min. At the end of the infusion (time zero), arterial blood samples were drawn at times 0, 15, 30, 60, 120 and 180 min. All samples were immediately refrigerated at 4 °C, and plasma was separated by centrifugation at 3000 rpm for 10 min and frozen at −80 °C within 24 h of sample collection.

#### 2.2.3. Lung Injury and ECMO Support

A 2-hit model of ARDS was applied as described in Araos et al. [25]. Briefly, lungs were depleted of alveolar surfactant by lavages with warm saline (30 mL·Kg^−1^, 39 °C), followed by injurious mechanical ventilation. The ECMO equipment included a magnetic Medtronic Bio-Medicus^®^ 540 centrifuged pump (Eden Praire, Chanhassen, MN, USA), a coagulation monitor (Hemochron^®^ Response, ITC, Hudsonville, MI, USA), and a heat exchanger HU-35 (Maquet, Wayne, NJ, USA). The circuit comprised a HILITE^®^ 2400LT polymethylpentene hollow fiber membrane oxygenator, 0.65 m^2^ (MEDOS, Stolberg, Germany), polyvinyl chloride ¼-inch lines coated with rheoparin, and a Rotaflow 32 head pump (Maquet, Wayne, NJ, USA). The circuit was primed with 250 mL solution saline. Veno-venous ECMO was applied through a 23 French bi-caval Avalon cannula inserted through the external right jugular vein [25].

#### 2.2.4. Timeframes of Meropenem Infusion during ECMO Support

**Figure 1:**
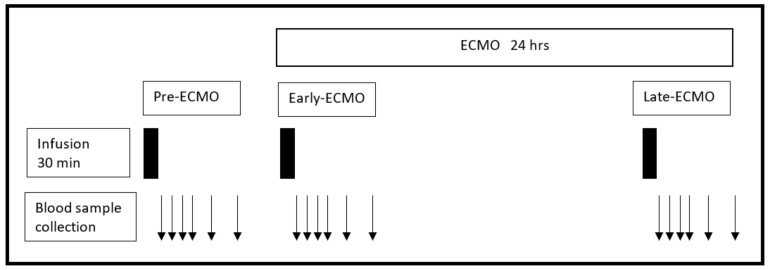
Schematic representation of the time points where blood sampling was performed. ECMO = Extracorporeal membrane oxygenation.

Meropenem was infused at 3 different time periods: (a) Pre-ECMO: prior to instrumenting the animals for ECMO support; (b) Early-ECMO, immediately after starting ECMO; and (c) Late-ECMO, 24 h after Early-ECMO (Figure 1).

### 2.3. Meropenem Measurements

#### Meropenem Measurement by the Biosensor PenP

The time-course of fluorescence measurements have been previously described [24]. Briefly, 10 μL of standard or conveniently diluted samples were placed into the wells of a black-walled 96-wells microplate. Then, they were mixed thoroughly with 190 μL of assay solution (5 × 10^−8^ M biosensor, with 1% (*w*/*v*) BSA in PBS buffer (pH 7.0)), and the fluorescence (em = 515 nm) was recorded through time by using a spectrofluorimeter Synergy2^®^ (Biotek, Winooski, VT, USA). We have previously observed that antibiotics resistant to lactamase activity induce a stable increase in the fluorescence level of the biosensor at any concentration. On the other hand, antibiotics that are sensitive to lactamase activity show a transient increase in the level of fluorescence at low concentrations, which strongly suggests the retention of residual catalytic activity in the biosenor. This disparity was solved by using the area under the curve (AUC) of the plotted fluorescence level against time induced by the different antibiotic concentrations. In this way, all the curves obtained were fitted to a 4PL or boltzman curve, independently of the antibiotic and its sensitivity to catalytic activity of the biosensor [24]. Thus, in this study the antibiotic concentration on each sample was interpolated into the AUC curve obtained from the standard curve (Figure S1 in Supplementary Materials). Each sample or standard measurement was carried out in triplicate and then reported as the mean ± standard error.

### 2.4. Pharmacokinetic Analysis

The pharmacokinetics of meropenem were adjusted to a mono-compartmental model, based on a single dose administered intravenously as an infusion. The volume of distribution (Vd) was calculated as follows: Vd = K_0_/Ke C_t_ (1 − e^−Ket^), where K_0_ is the perfusion constant, Ke is the elimination rate constant, Ct is the concentration at a time t and t is any time considered. The value of Ke was calculated from the slope of the equation of the line, obtained after the linearization of the descending mono-exponential curve of the concentrations of the blood samples collected at different times, once the perfusion was completed.

### 2.5. Statistical Analysis

Statistical analysis was performed using SPSS for Windows NT v.13.0 (SPSS Inc., Chicago, IL, USA). Descriptive statistics were computed for all study variables. A *p*-value < 0.05 was considered statistically significant.

## 3. Results

### Pharmacokinetics of Meropenem during ECMO

**Figure 2:**
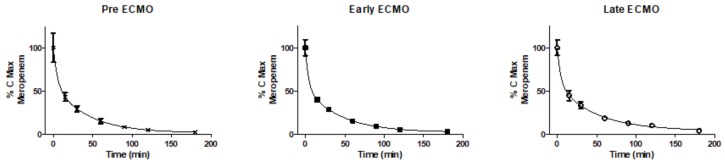
Curves showing the maximum concentration (Cmax) of meropenem (%) versus time. Maximum concentration of meropenem was 131 ± 59 M, 154 ± 52 M and 137 ± 27 M for Pre-ECMO, Early ECMO and late ECMO, respectively.

In order to validate the data obtained by the biosensor PenP, solutions of meropenem at different concentration (130–6500 uM) were determined by HPLC and then compared against those values obtained by PenP (Figure S2 in Supplementary Materials). Results from both methods showed an excellent lineal symmetry (*R*^2^ = 0.9945) in the range analyzed, indicating the accuracy of PenP values respect to the gold-standard technique. The plasma concentration of meropenem along the time were quiet similar among the three conditions studied (pre-ECMO, ECMO-early and ECMO-late) Figure 2. The kinetics of meropenem showed a clearance of more than 50% during the first 15 min and a long plateau phase after one and half hours. The plasmatic meropenem concentration curves, normalized as a percentage of the maximum concentration, were adjusted to an exponential decay curve of two phases in each conditions studied (pre-ECMO, ECMO-early and ECMO-late) (*R*^2^ > 0.9). The concentration of meropenem at the same time points did not show statistically significant differences among the three periods. Consequently, the PK parameters derived from the curves, for each condition, assuming a mono-compartmental model, did not show significant differences in the elimination rate constant (Ke), clearance or Vd (Table 1).

## 4. Discussion

Our results show that the biosensor PenP accurately measures a wide range of meropenem concentrations, similar to HPLC, which is considered the gold-standard. The main advantage of this biosensor, compared to the HPLC, lies on its parallel data collection structure. For example, the time required to process 96 samples by HPLC, given its sequential structure, would imply approximately 16 h, whereas the reported method based on parallel fluorescence readings of PenP allows for analysis of the same sample volume in only 30 min. Importantly, we have demonstrated that the technique based on PenP is easy to implement and thus may be used clinically. In this context, the technique shows promise regarding its potential use to help provide personalized dosing, especially during care of critically ill patients.

In this model of severe ARDS, we have shown that the PK parameters of meropenem (Vd, Ke and Cl) do not seem to be affected by the institution of ECMO, as these values were similar before, during and 24 h after starting ECMO support. Thus, despite the potential for ECMO to affect elimination and degradation or to produce circuit sequestration of antibiotics, our results suggest that at least during the timeframe studied, instrumental absorption of meropenem is not clinically significant.

## 5. Conclusions

Pen-PC can be used as a fast and accurate tool to measure meropenem concentrations through a wide range of clinically relevant concentrations. In a 24-h animal model of severe ARDS, ECMO support does not seem to significantly affect the pharmacokinetics of intravenously perfused meropenem.

## Figures and Tables

**Table 1:** Pharmacokinetics of meropenem at different periods. Cmax: maximum concentration; Cl: clearance of meropenem and Vd: volume of distribution. Values are expressed as mean ± SEM ANOVA test * *p* > 0.05.

	Pre-ECMO (n = 3)	Early ECMO (n = 5)	Late ECMO (n = 5)
Cmax (umol/Kg)	2.196 ± 0.877	2.507 ± 0.677	2.311 ± 0.567
Cl (L/min/Kg)	11.92 ± 3.93	8.96 ± 1.84	9.26 ± 1.69
Ke (min^−1^)	0.024 ± 0.007	0.020 ± 0.003	0.019 ± 0.002
Vd (L/Kg)	497 ± 134	448 ± 92	490 ± 89

## Supplementary Materials

The following are available online at http://www.mdpi.com/1424-8220/18/5/1424/s1, Figure S1: Analysis Method: (a) data collection; (b) subtraction of background; (c) amplitude of arbitrary fluorescence; and (d) fiting of AUCs to 4PL curve. Figure S2: (a) Data collection; (b) amplitude of arbitrary fluorescence; (c) interpolation of AUCs into standard 4PL curve; and (d) concentration determination. Figure S3: Comparison of the concentration value determined by PenP with respect to HPLC. Table S1 Values of concentration determined by PenP with respect to HPLC.
