# Supplementary material for: Evaluation of Meropenem Pharmacokinetics in an Experimental Acute Respiratory Distress Syndrome (ARDS) Model during Extracorporeal Membrane Oxygenation (ECMO) by Using a PenP β-Lactamase Biosensor
Source: Sensors (Basel). 2018 May 4;18(5):1424. doi: 10.3390/s18051424 (PMC5982397; doi:10.3390/s18051424)
Supplement: Supplementary file 1 [file sensors-18-01424-s001.pdf]

sensors

MDPI

Methods

1. Meropenem Measurement by PenPc

2.1. Standard Curve

A standard curve was carried out as previously described [1]. Briefly, 10 μL of meropenem solution at different concentrations (from 10^{-8} to 10^{-4} M) prepared in PBS were placed in a black-walled 96-well microplate. Next, 190 μL/well of biosensor solution (5×10^{-8} M) was added to each well and mixed thoroughly. The fluorescence was then recorded as a function of time.

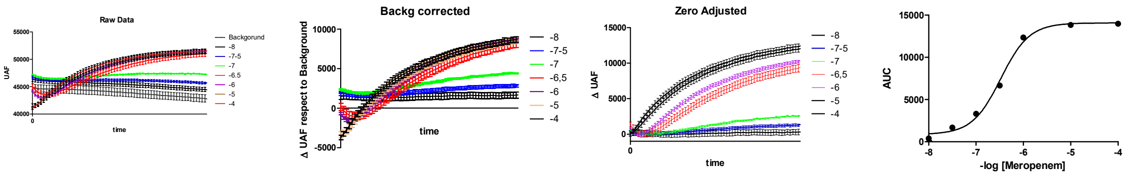

(**a**) (**b**) (**c**) (**d**)

Figure S1. Analysis Method: (**a**) data collection; (**b**) subtraction of background; (**c**) amplitude of arbitrary fluorescence; and (**d**) fitting of AUCs to 4PL curve.

2.2. Samples Interpolation

Serial dilutions (PBS) of plasma samples from 1:1 to 1:1000 were performed. Ten microliters of each dilution were placed in a black-walled 96-well microplate. Next, 190 μL/well of biosensor solution (5×10^{-8} M) was added to each well and mixed thoroughly. The fluorescence was then recorded as a function of time. From the zero-adjusted curves, the AUC obtained from the first non-saturating dilution data was interpolated in the 4PL standard curve and this concentration was multiplied by the dilution factor in order to determine the Meropenem concentration.

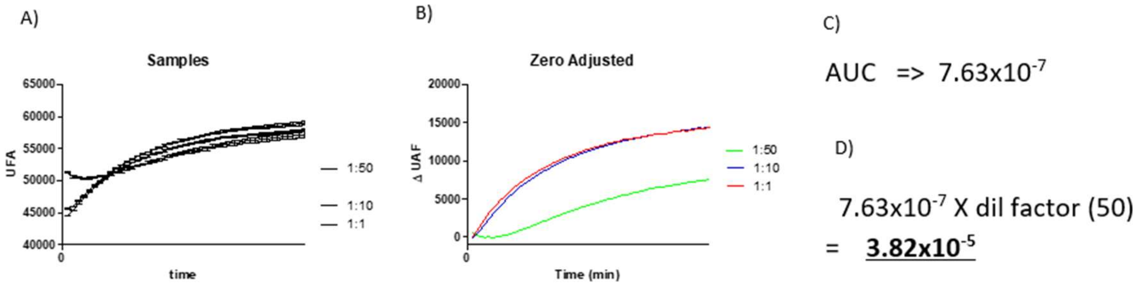

Figure S2. (**a**) Data collection; (**b**) aAmplitude of arbitrary fluorescence; (**c**) interpolation of AUCs into standard 4PL curve; and (**d**) concentration determination.

2. Meropenem Measurement by HPLC

The HPLC protocol to determine Meropenem has been previously described [2]. Briefly, an Agilent 1200 series equipped with diode array detector (DAD) and u-Bondpack C18 (3.9 × 300 mm) column was used. The isocratic mobile phase consisted of 21.2% acetonitrile, 78% H_2O and 0.8% glacial acetic acid, and the flow rate was 1.2 mL/min. Meropenem from control plasma was extracted by mixing the plasma with equal volume (0.5 mL) of trichloroacetic acid solution. The mixture was shaken for 30 s followed by centrifugation for 5 min at 3000 rpm. Aliquots of 15 uL were injected onto the column and the effluent was monitored at 296 nm. The areas under the curve (AUC) of meropenem were normalized against the AUC of the internal standard pheniramine. Each

concentration of standard samples determined by HPLC was compared against the concentration determinate by PenPc method. Determinations were carried out at least in triplicate and then reported as the mean ± standard error (Figure S3 and Table S1).

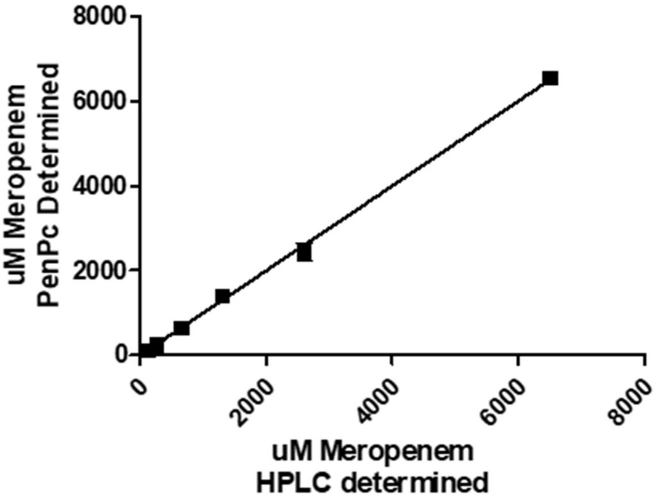

Figure S3. Comparison of the concentration value determined by PenP with respect to HPLC.

Table S1. Values of concentration determined by PenP with respect to HPLC.

HPLC STD (uM)	PenP Determined (uM)	SEM	n
130	113	10	6
260	241	47	3
650	640	4	3
1300	1398	49	6
2600	2444	201	3
6500	6537	103	3
